# Neuroprotective effects of alpha-pinene against behavioral deficits in ketamine-induced mice model of schizophrenia: Focusing on oxidative stress status

**DOI:** 10.1016/j.ibneur.2023.12.012

**Published:** 2024-01-04

**Authors:** Akbar Hajizadeh Moghaddam, Fatemeh Malekzadeh Estalkhi, Sedigheh Khanjani Jelodar, Tabarek Ahmed Hasan, Soroush Farhadi-Pahnedari, Mohammad Karimian

**Affiliations:** a Department of Animal Biology, Faculty of Basic Sciences, University of Mazandaran, Babolsar, Iran; b Faculty of Biotechnology, Amol University of Special Modern Technologies, Amol, Iran; c Department of Molecular and Cell Biology, Faculty of Basic Sciences, University of Mazandaran, Babolsar, Iran

**Keywords:** Schizophrenia, Alpha-pinene, Ketamine, Oxidative stress, Mice

## Abstract

Schizophrenia (SCZ) is a profound neurological disorder that affects approximately 1% of the global population. Alpha-pinene (α-pinene) is a natural and active monoterpene found in coniferous tree oil, primarily pine, with diverse pharmacological characteristics, including antioxidative, anxiolytic, and antidepressant properties. This research study delves into the neuroprotective effects of α-pinene on oxidative stress, memory deficits, and depressive and anxiety-like behaviors in a ketamine-induced mice model of SCZ using male mice. The mice were randomly divided into six groups: vehicle, control, positive control, ketamine, α-pinene at 50 mg/kg, and α-pinene at 100 mg/kg. Treatment of the ketamine-induced mice model of SCZ with α-pinene yielded significant improvements in depressive and anxiety-like behaviors and cognitive impairments. Furthermore, it significantly elevated glutathione (GSH) levels, total antioxidant capacity (TAC), dopamine levels, catalase (CAT), and superoxide dismutase (SOD) activities while markedly reducing malondialdehyde (MDA) levels. The current study establishes that α-pinene treatment effectively mitigates oxidative damage, cognitive deficits, and depressive and anxiogenic-like behaviors in the brains of ketamine-treated mice. Therefore, α-pinene treatment is an efficacious approach to forestall the neurobehavioral and neurobiochemical adverse effects of the ketamine-induced SCZ model of mice.

## 1 Introduction

Schizophrenia (SCZ) is a chronic mental disorder that affects approximately 1% of the global population (Rabinovitch et al., 2023, Réus et al., 2017; Venkataramaiah et al., 2021). Its symptoms can be characterized by negative symptoms (e.g., depressive and anxiety-like behaviors), positive symptoms (e.g., disorganized speech, hallucinations, and unusual thoughts), and cognitive symptoms (e.g., memory-associated dysfunctions and inattention) (Chatterjee and Chatterjee, 2023, Temmingh and Stein, 2015, Yolland et al., 2020). SCZ typically emerges at the end of adolescence, and its course is generally marked by alternating episodes of disorder exacerbation and partial remission (Kegeles et al., 2010, Namba and Nawa, 2020, Zoghbi et al., 2020).

Studies have indicated that dopaminergic hyperactivity in the brain is associated with the manifestation of SCZ symptoms, particularly psychosis (Kokkinou et al., 2021). Additionally, oxidative stress and redox imbalance play a significant role in the pathogenesis of this mental disorder (Ermakov et al., 2021). Anxiety symptoms are prevalent among most SCZ patients, adversely affecting the course of the condition (Temmingh and Stein, 2015). Research shows SCZ symptoms like depression and memory loss are directly linked to increased oxidative stress (Ben-Azu et al., 2018b).

Conversely, mitochondrial dysfunction, abnormalities in ATP production, and calcium buffering disturbances contribute to reduced metabolism and synaptic activity in SCZ (Maguire et al., 2020, Roberts, 2017). It has been observed that excessive production of reactive oxygen species (ROS) leads to detrimental effects such as lipid peroxidation, which damages neurons, including parvalbumin-expressing (PV+) GABAergic interneurons, resulting in decreased inhibitory tone in cortical and hippocampal brain regions (Ben-Azu et al., 2018a, Ben-Azu et al., 2022). In conditions related to oxidative stress, the function of dopamine receptors is damaged (Zeng et al., 2009). Increased levels of dopamine, causes increased oxidative stress, depletion of glutathione, and resulting decreases in tissue GABA concentrations (Lyng and Seegal, 2008).

Neuropharmacological studies have also demonstrated that non-competitive N-methyl-D-aspartate (NMDA) receptor antagonists, including phencyclidine (PCP) and ketamine, induce SCZ-like symptoms such as thought disturbances, unusual ideas, illusions, cognitive impairment, and emotional withdrawal (Hsieh et al., 2021; Venkataramaiah et al., 2021). Ketamine hydrochloride, a derivative of PCP, was initially synthesized in 1962. Ketamine is utilized to model SCZ by acting on the glutamate system (Beck et al., 2020). Additionally, studies have demonstrated that ketamine stimulates dopamine release (Morgan and Curran, 2012, Omeiza et al., 2023). Ketamine can alter GABAergic neurons, causing a hyperdopaminergic state. This links to increased activity and repetitive behaviors, related to psychosis symptoms (Yadav et al., 2018b). It has also been reported to increase lipid peroxidation and alter antioxidant enzyme activity, leading to oxidative stress (Ben-Azu et al., 2018b, Yadav et al., 2018c).

Alpha-pinene (α-pinene) is a natural and active monoterpene found in pine oil conifer oil. It finds application in various industries, including pharmaceuticals, flavorings, perfumes, and even as a raw material in jet fuel (Yang et al., 2013, Bouzenna et al., 2017). In plants, α-pinene synthesis occurs through the head-to-tail condensation of dimethylallylpyrophosphate (DMAPP), isopentenyl pyrophosphate (IPP), and the precursor geranyl pyrophosphate (C10) (Allenspach and Steuer, 2021). Today, herbal essential oils are employed in traditional medicine to treat mental disorders such as depression, anxiety, neurosis, and bipolar disorder. Furthermore, according to several studies, α-pinene possesses anti-inflammatory, anticonvulsant, bactericidal, hypnotic, analgesic, anti-tumor, and antioxidant properties (Rahbar et al., 2019, Ueno et al., 2019). In this context, it has been demonstrated that α-pinene exerts a neuroprotective effect in ischemic stroke in rats due to its antioxidant and anti-inflammatory functions (Khoshnazar et al., 2019). Additionally, other research confirms that α-pinene, through its antioxidant effects on cardiac markers, reduces lipid peroxidation, safeguards the heart myocardium, and exhibits cardioprotective and anti-inflammatory effects in rats (Zhang et al., 2020). Therefore, the study investigates the effects of α-pinene on antioxidant parameters and behavioral changes in a ketamine-induced SCZ-like mice model.

## 2 Materials and methods

### 2.1 Animals

Sixty male mice (30–35 g) were obtained from the Pasteur Institute (Amol, Iran) and transferred to the animal care center of the Biology Department at the University of Mazandaran, Babolsar, Iran. The animals were given unrestricted access to standard laboratory food and water, maintained at a temperature of 23 ± 2 °C, with a humidity level of 50%, and subjected to a 12-hour light/dark cycle. Furthermore, they underwent a seven-day acclimatization period in the laboratory environment before the commencement of the research. The experiment adhered to ethical guidelines approved by the University of Mazandaran Ethics Committee (IR.UMZ.REC.1401.063). All chemicals employed in this study were procured from Sigma-Aldrich, Alfasan, and Merck. Additionally, α-pinene, used for synthesis, was sourced from Sigma Aldrich with the registration number 8.18405.0100.

### 2.2 Experimental design

**Fig. 1:**
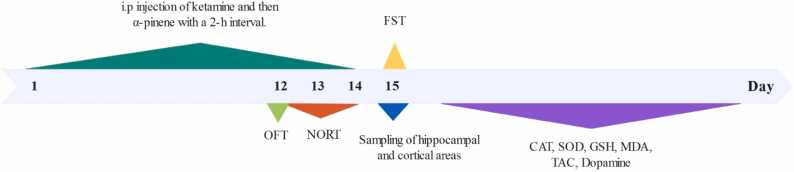
Timeline of the experimental method.

Animals were randomly divided into (Hsieh et al., 2021) 6 groups:

1. **Control:** Animals in this group did not receive any treatment.
2. **Vehicle:** Animals received a gavage of Tween 80 and saline.
3. **Positive control:** Animals received a gavage of α-pinene at 50 mg/kg.
4. **KET:** Animals were injected with ketamine at 20 mg/kg i.p.
5. **KET + α-pinene 50:** Animals were injected with ketamine at 20 mg/kg i.p. After 2 h, they received α-pinene at 50 mg/kg orally.
6. **KET + α-pinene 100:** Animals were injected with ketamine at 20 mg/kg i.p., and after 2 h, they received α-pinene at 100 mg/kg orally (Fig. 1).

Saline was used as the solvent for ketamine, and a mixture of Tween 80 and saline was used as the solvent for α-pinene (Khoshnazar et al., 2019, Moghaddam et al., 2021). All treatments were administered for 14 consecutive days. The animal model of SCZ-like was induced by injecting ketamine at 20 mg/kg.

### 2.3 Behavioral assessment

All animals during the last 3 days of the 14-day treatment period, and 3 h after the last treatment, were randomly assessed for behavioral analyses. The same animals were present throughout the tests. At first, OFT was performed on the 12th day and NORT was conducted on the 12th, 13th, and 14th days, and then FST was performed on day 15 (Fig. 1).

#### 2.3.1 OFT (Open Field Test)

In this test, animals were placed in the center area of the apparatus and allowed to explore freely for 10 min. Animal activities were recorded during the time spent in the center area to assess anxiety-like behaviors (Choleris et al., 2001).

#### 2.3.2 FST (Forced Swimming Test)

The FST was utilized to evaluate depressive-like behaviors. Animals were individually placed in a glass cylinder filled with water (15 cm from the bottom) at 25 °C, with dimensions of 30 cm in height and 20 cm in diameter. Swimming and immobility times were recorded over 5 min (Ben-Azu et al., 2018a).

#### 2.3.3 NORT (Novel Object Recognition Test)

The NORT was employed to assess learning and memory function in the animals. The test consisted of three sequential sessions conducted daily, each lasting 5 min:

1. **Habituation Day:** Animals explored an empty box to adapt to the environment.
2. **Teaching Day:** Animals were exposed to two identical objects.
3. **Test Day:** Animals encountered one familiar and one novel object. The exploration time, defined as smelling or touching the objects in the box, was measured to evaluate recognition memory (Yuede et al., 2009). At the end of the test, the discrimination index was calculated as:

$$\mathrm{Discrimination Index}(\mathrm{DI})=\frac{\mathrm{Novel Object Exploration Time}}{\mathrm{Total Exploration Time}}\times 100$$

### 2.4 Biochemical parameters assay

#### 2.4.1 Tissue preparation

After the behavioral tests, all animal groups were euthanized on the 15th day, and the entire cortex and hippocampal area were extracted and homogenized with 1 ml of saline phosphate buffer (containing 0.32 mol/l sucrose, 1 mmol/l EDTA, and 10 nmol/l Tris-HCL, pH=7.4). The homogenized tissues were centrifuged for 30 min at 12,000 rpm and 4 °C. Subsequently, the supernatant was separated and stored at − 80 °C.

#### 2.4.2 Evaluation of CAT and SOD activities

Oxidative stress parameters were assessed, including CAT and SOD activities. CAT activity was determined using Aebi's method (Aebi, 1974). The reaction mixture consisted of H_2_O_2_ and phosphate buffer (475 μl, pH 7.0), and the amount of H_2_O_2_ decomposition was measured at 240 nm. SOD activity was assessed based on the method described by McCord and Fridovich, which quantifies the enzyme's ability to prevent pyrogallol autoxidation to half-maximum (McCord and Fridovich, 1969).

#### 2.4.3 Measurement of GSH and MDA levels

GSH levels were measured using Ellman's procedure (Ellman, 1959). This estimation is based on the reaction of GSH with Ellman's reagent (DTNB or 5,5-dithio-bis-(2-nitrobenzoic acid)). The formation of TNB (2-Nitro-5-mercapto-benzoic acid) in the sample was evaluated at a wavelength of 412 nm, proportional to the extent of glutathione present. The methods of Esterbauer and Cheeseman were employed to assess lipid peroxidation. MDA levels were measured at 535 nm as a marker of lipid peroxidation (Esterbauer and Cheeseman, 1990).

#### 2.4.4 Total antioxidant capacity (TAC)

TAC was determined according to the procedure proposed by Benzie and Strain (Benzie and Strain, 1996). This method reduces Fe^3+^ to Fe^2+^ in the presence of 2,4,6-Tris(2-pyridyl)S-triazine (TPTZ). In this study, the standard FeSO_4_.7 H_2_O was used to assay the total antioxidants in the samples. The assay mixture contained 0.3 M sodium acetate buffer (pH = 3), 10 mM TPTZ, 20 M ferric chloride solution, and 50 ml of tissue supernatant, resulting in a total volume of 1.5 ml. The absorbance was measured at 593 nm after incubating for 10 min at 30 °C.

#### 2.4.5 Assessment of dopamine levels

Dopamine levels were measured using Li Guo’s method, based on reducing Fe (III) to Fe (II) by dopamine, and measured colorimetrically at a wavelength of 735 nm (Guo et al., 2009). Specifically, 1 ml of the sample was mixed with 1 ml of ferric chloride and potassium ferricyanide, and the volume was adjusted to 25 ml with distilled water. The mixture was allowed to stand at room temperature for 35 min and evaluated with a spectrophotometer. The dopamine levels in the samples were reported as ng/mg protein (Jolodar et al., 2020).

#### 2.4.6 Evaluation of protein content

Protein content in the samples was determined using Bradford's method, with bovine serum albumin (BSA) serving as the standard (Bradford, 1976).

### 2.5 Statistical analysis

The results were presented as mean ± SD and analyzed using one-way ANOVA followed by Tukey's post-hoc test. A significance level of *P* < 0.05 was considered statistically significant.

## 3 Results

### 3.1 Effects of α-pinene on depressive- and anxiety-like behaviors

**Fig. 2:**
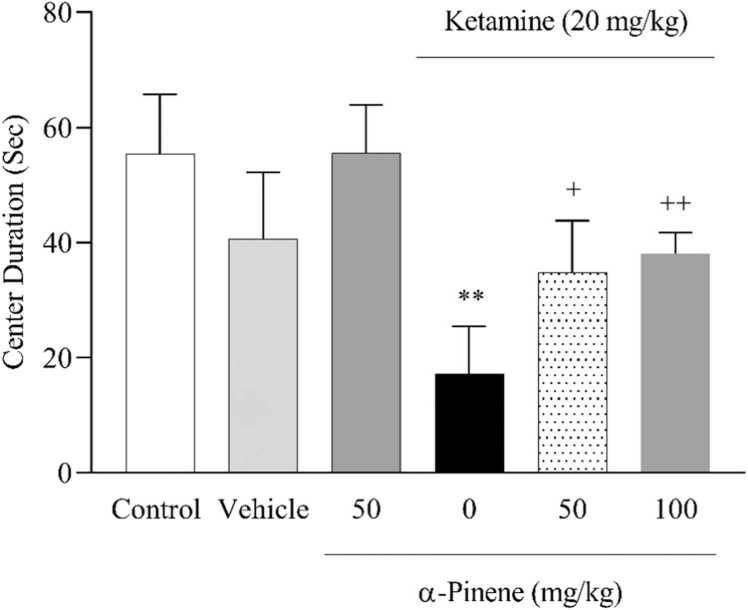
Effects of α-Pinene on anxiety-like behaviors in OFT. Values are means ± S.D. Tukey’s Post hoc test was used to compare between groups. * * *P* < 0.01, vs. vehicle group. + *P* < 0.05 and + + *P* < 0.01, vs. KET group.

Results from OFT analyses (Fig. 2) demonstrated that center duration in the KET group significantly decreased (*P* < 0.01) compared to the vehicle group [F (5, 24) = 12.96, *P* < 0.001]. This period in α-pinene groups at 50 mg/kg and 100 mg/kg doses significantly increased (*P* < 0.05) and (*P* < 0.01) respectively, compared to the KET group. Behavioral analyses in FST (Fig. 3.1) showed that immobility time in the KET group was significantly enhanced (*P* < 0.05) compared to the vehicle group [F (5, 35) = 7.08, *P* < 0.001]. Immobility time in the α-pinene group at 100 mg/kg was reduced significantly (*P* < 0.01) compared to the KET group. In continuation, swimming duration (Fig. 3.2) in the KET group was significantly reduced (*P* < 0.001) compared to the vehicle group [F (5, 30) = 8.56, *P* < 0.001], and in both doses of α-pinene was increased significantly (*P* < 0.05) compared to the KET group.

**Fig. 3:**
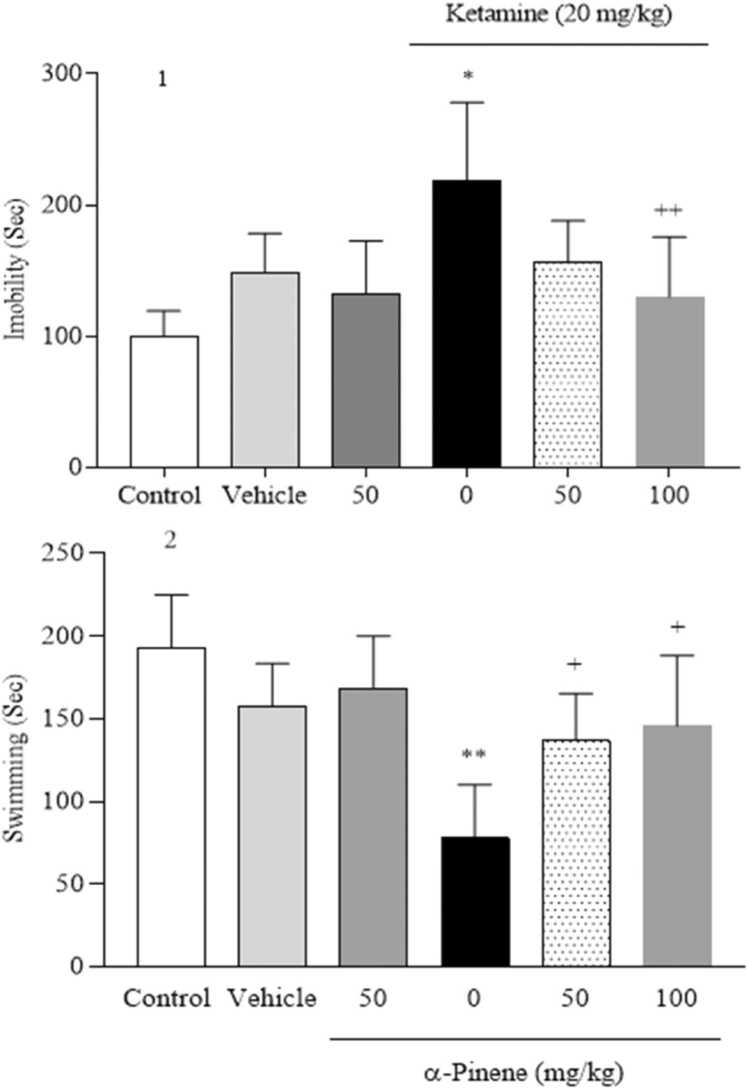
Effects of α-Pinene on depressive-like behaviors in FST. Values are means ± S.D. Tukey’s Post hoc test was used to compare between groups. * *P* < 0.05 and * * *P* < 0.01, vs. vehicle group. + *P* < 0.05 and + + *P* < 0.01, vs. KET group.

### 3.2 Effects of α-pinene on cognitive deficits

**Fig. 4:**
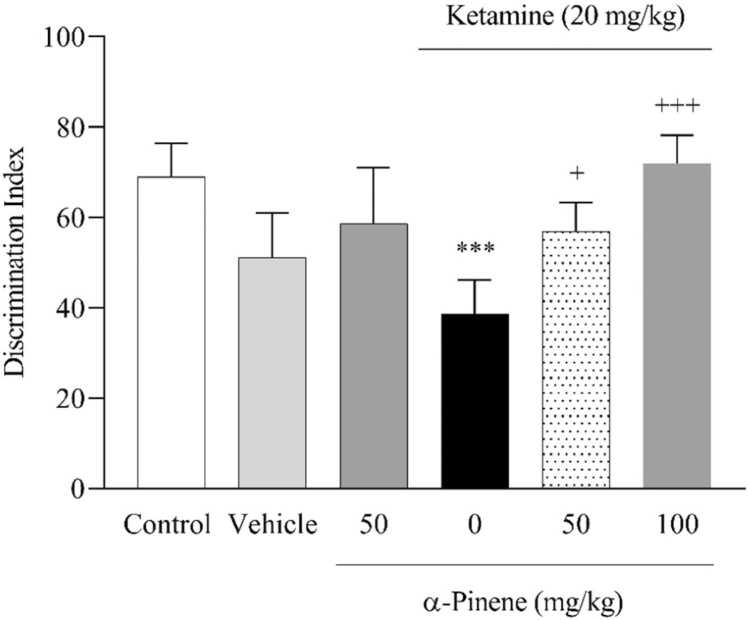
Effects of α-Pinene on memory deficits in the NORT. Values are means ± S.D. Tukey’s Post hoc test was used to compare between groups. * ** *P* < 0.001, vs. vehicle group. + *P* < 0.05 and + ++ *P* < 0.001, vs. KET group.

In the study of NORT results (Fig. 4), it was shown that the discrimination index [F (5, 24) = 7.33, *P* < 0.001] in the KET group significantly decreased compared to the vehicle group. The α-pinene group at 50 mg/kg and 100 mg/kg significantly increased the discrimination index compared to the KET group (*P* < 0.05) and (*P* < 0.001) respectively.

**Fig. 5:**
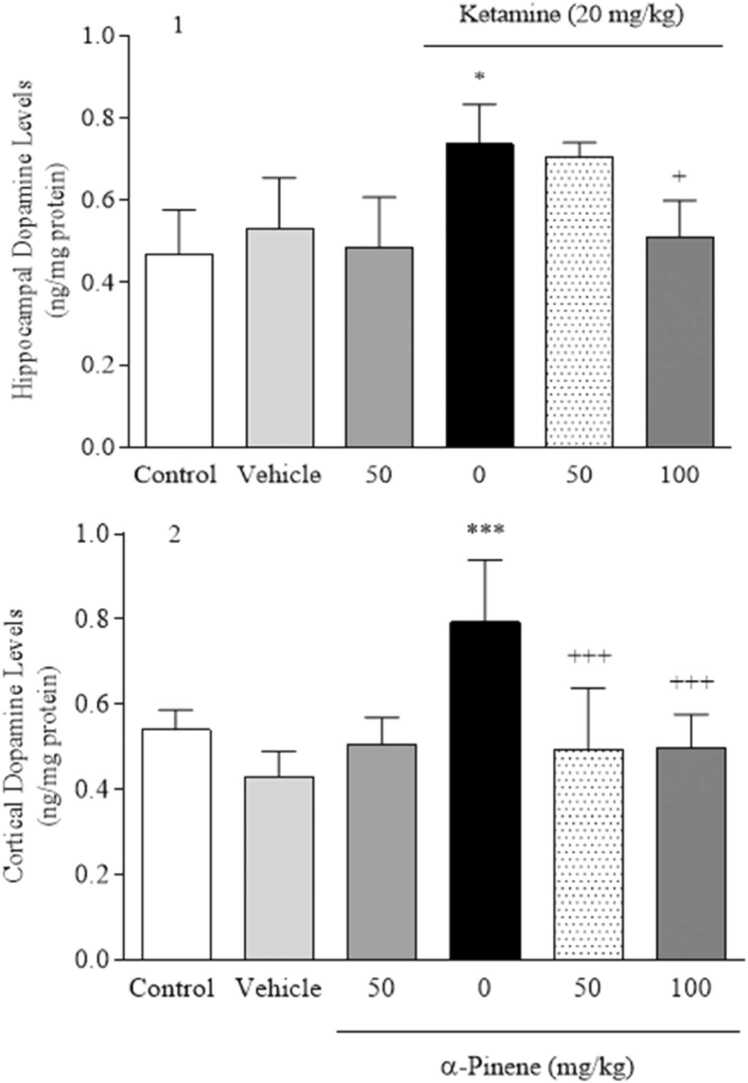
Effects of α-Pinene on dopamine levels. Values are means ± S.D. Tukey’s Post hoc test was used to compare between groups. * *P* < 0.05 and * ** *P* < 0.001 vs. vehicle group. + *P* < 0.05 and + ++ *P* < 0.001 vs. KET group.

### 3.3 Effects of α-pinene on CAT and SOD activities

Cortical CAT activity (Table 1) in the KET group significantly reduced (*P* < 0.05) compared to the vehicle group [F (5, 24) = 7.53, *P* < 0.001]. Whenever α-pinene groups at 50 and 100 mg/kg significantly reversed cortical CAT activity (*P* < 0.05) and (*P* < 0.01) respectively compared to the KET group. Hippocampal CAT activity in the KET group significantly reduced (*P* < 0.05) compared to the vehicle group [F (5, 24) = 7.51, *P* < 0.001]. While the α-pinene group at 100 mg/kg significantly reversed hippocampal CAT activity (*P* < 0.05) compared to the KET group. Also, the α-pinene group at 100 mg/kg significantly increased hippocampal CAT activity (*P* < 0.05) compared to the group of α-pinene at 50 mg/kg. After 14 consecutive days of ketamine injection, we observed a significant reduction in SOD activity (Table 1) in the hippocampus [F (5, 24) = 10.39, *P* < 0.001] (*P* < 0.001) and cortex [F (5, 24) = 6.55, *P* < 0.01] (*P* < 0.05) compared to the vehicle group. Whereas the α-pinene group at 100 mg/kg significantly reversed (*P* < 0.05) hippocampal SOD activity compared to the KET group. Also, α-pinene groups at both doses significantly reversed (*P* < 0.01) cortical SOD activity compared to the KET group.

**Table 1:** Effects of α-Pinene on antioxidant enzyme activities in ketamine-induced schizophrenia model.

Hippocampus		
Groups	CAT (U/mg protein)	SOD (U/mg protein)
Control	165.36 ± 29.42	85.53 ± 10.71
Vehicle	145.99 ± 20.82	81.58 ± 14.39
Positive control	130.67 ± 34.90	77.84 ± 10.14
KET (20 mg/kg)	78.31 ± 16.78 *	36.23 ± 13.59 * **
KET + α-Pinene (50 mg/kg)	77.03 ± 13.35	56.59 ± 15.52
KET + α-Pinene (100 mg/kg)	137.38 ± 24.77 + #	66.89 ± 12.11 +
**Cortex**		
Control	145.47 ± 30.64	63.16 ± 8.34
Vehicle	113.15 ± 24.16	62.57 ± 8.18
Positive control	114.49 ± 30.08	74.90 ± 10.12
KET (20 mg/kg)	53.46 ± 23.16 *	37.12 ± 13.96 *
KET + α-Pinene (50 mg/kg)	106.10 ± 10.46 +	67.70 ± 11.18 + +
KET + α-Pinene (100 mg/kg)	121.41 ± 24.68 + +	65.51 ± 14.20 + +

Data are reported as the mean ± SD of five male mice in each group. Tukey’s Post hoc test was used to compare between groups. * P < 0.05 and * ** P < 0.001 as compared to the vehicle group. + P < 0.05 and + + P < 0.01 as compared to KET group. # P < 0.05 as compared to the (KET + α-pinene 50 mg/kg) group.

### 3.4 Effects of α-pinene on GSH and MDA levels

GSH evaluation (Table 2) revealed that glutathione levels in the KET group significantly decreased compared to the vehicle group in hippocampal (*P* < 0.01) [F (5, 24) = 9.64, *P* < 0.001] and cortical areas (*P* < 0.001) [F (5, 24) = 21.70, *P* < 0.001]. Treatment with α-pinene at both doses significantly increased hippocampal GSH levels (*P* < 0.01) compared to the KET group. Cortical GSH levels significantly increased (*P* < 0.001) with α-pinene 100 mg/kg treatment compared to the KET group. Also, cortical GSH levels with α-pinene treatment at 100 mg/kg showed a significant increase compared to 50 mg/kg (*P* < 0.001). As well, our data showed that hippocampal [F (5, 24) = 12.99, *P* < 0.001] and cortical [F (5, 24) = 25.43, *P* < 0.001] MDA levels (Table 2) significantly increased (*P* < 0.001) in the KET group compared to the vehicle group. While α-pinene groups at 50 and 100 mg/kg significantly decreased hippocampal MDA levels (*P* < 0.01) compared to the KET group. The groups of α-pinene at both doses significantly decreased cortical MDA levels (*P* < 0.001) compared to the KET group.

**Table 2:** Effects of α-Pinene on MDA, TAC, and GSH levels in ketamine-induced schizophrenia model.

Hippocampus			
Groups	MDA (µg/mg protein)	TAC (mg/gr protein)	GSH (mg/gr protein)
Control	0.23 ± 0.03	0.13 ± 0.01	0.38 ± 0.03
Vehicle	0.31 ± 0.03	0.14 ± 0.02	0.31 ± 0.07
α-Pinene 50	0.29 ± 0.05	0.13 ± 0.02	0.34 ± 0.03
KET (20 mg/kg)	0.51 ± 0.08 * **	0.04 ± 0.02 * **	0.17 ± 0.04 * *
KET + α-Pinene (50 mg/kg)	0.34 ± 0.08 + +	0.11 ± 0.01 + ++	0.29 ± 0.03 + +
KET + α-Pinene (100 mg/kg)	0.33 ± 0.03 + +	0.15 ± 0.01 + ++	0.33 ± 0.06 + +
**Cortex**			
Control	0.31 ± 0.02	0.047 ± 0.011	0.4 ± 0.02
Vehicle	0.26 ± 0.03	0.027 ± 0.007	0.34 ± 0.07
α-Pinene 50	0.28 ± 0.01	0.037 ± 0.011	0.38 ± 0.06
KET (20 mg/kg)	0.51 ± 0.08 * **	0.006 ± 0.002 *	0.17 ± 0.04 * **
KET + α-Pinene (50 mg/kg)	0.27 ± 0.004 + ++	0.038 ± 0.009 + ++	0.2 ± 0.04
KET + α-Pinene (100 mg/kg)	0.27 ± 0.04 + ++	0.03 ± 0.008 + +	0.39 ± 0.01 + ++ ###

Data are reported as the mean ± SD of five male mice in each group. Tukey’s Post hoc test was used to compare between groups. * *P* < 0.05, * * *P* < 0.01, and * ** *P* < 0.001 as compared to the vehicle group. + + *P* < 0.01 and + ++ *P* < 0.001 as compared to KET group. ### *P* < 0.001 as compared to the (KET + α-pinene 50 mg/kg) group.

### 3.5 Effects of α-pinene on total antioxidant capacity

The results of this study showed that TAC (Table 2) in the KET group had a significant reduction compared to the vehicle group in the hippocampal [F (5, 24) = 20.36, *P* < 0.001] (*P* < 0.001) and cortical [F (5, 24) = 12.56, *P* < 0.001] (*P* < 0.05) areas respectively. Whereas hippocampal antioxidant capacity in the group of α-pinene at both doses significantly increased (*P* < 0.001) compared to the KET group. Cortical antioxidant capacity in the group of α-pinene at 50 mg/kg and 100 mg/kg significantly increased (*P* < 0.001) and (*P* < 0.01) respectively compared to the KET group.

### 3.6 Effects of α-pinene on dopamine levels

The results of the examination showed that hippocampal dopamine levels (Fig. 5.1) in the KET group showed a significant increase (*P* < 0.05) compared to the vehicle group [F (5, 24) = 6.24, *P* < 0.01]. Whereas the α-pinene group at 100 mg/kg significantly decreased (*P* < 0.05) compared to the KET group. Also, cortical dopamine levels (Fig. 5.2) in the KET group increased significantly (*P* < 0.001) compared to the vehicle group [F (5, 24) = 8.45, *P* < 0.001]. While the group of α-pinene at both doses significantly decreased (*P* < 0.001) compared to the KET group.

## 4 Discussion

According to the current research, treatment with α-pinene significantly improved cortical and hippocampal antioxidant parameters, as well as cognitive impairments, anxiety, and depressive-like behaviors in a ketamine-induced SCZ model of mice. Ketamine, a non-competitive NMDAR antagonist, temporarily induces acute SCZ-like symptoms and is commonly used to model the condition (Frohlich and Van Horn, 2014). NMDAR deficiency leads to the downregulation of antioxidant genes and cortical circuit inhibition, generating ROS. These events may lead to oxidative damage and glutathione depletion, suppressing NMDAR function. These cumulative effects during development can result in alterations in cognition, behavior, and sensory processing associated with SCZ (Hardingham and Do, 2016).

Results from the OFT indicated that ketamine administration induced anxiety-like behaviors, but treatment with α-pinene at both doses reversed these anxiety-like behaviors in the ketamine-treated mice. Furthermore, the FST results revealed that the ketamine-induced mice model of SCZ led to the development of depressive-like behaviors, which were significantly reduced in the ketamine-induced SCZ model in mice following treatment with α-pinene at a dose of 100 mg/kg. Several studies have highlighted α-pinene's substantial anxiolytic properties as a bicyclic monoterpene (Ueno et al., 2019, Rahbar et al., 2019, Salehi et al., 2019). Additionally, research has demonstrated the efficacy of α-pinene in alleviating cognitive deficits and reducing anxiety and depressive-like behaviors. These studies have shown that α-pinene treatment reduces immobility in the FST and improves depressive-like behavior. Furthermore, α-pinene treatment has been associated with improvements in behavioral abnormalities, likely linked to enhanced oxidative phosphorylation and increased parvalbumin mRNA expression in the hippocampal and cortical regions (Kong et al., 2017). These findings suggest that α-pinene affects GABAergic signaling, potentially contributing to its antidepressant effects. The overall results indicate that α-pinene exerts its antidepressant and anxiolytic effects through 5-HT1A, β-Adrenergic, and D1 receptors while also enhancing hippocampal BDNF and dopamine synthesis, both of which are critical factors in the pathogenesis of depression and anxiety (Weston-Green et al., 2021).

The present research demonstrated that treatment with α-pinene at both doses improved cognitive deficits in the ketamine-induced SCZ model of mice in the NORT. Notably, the 100 mg/kg dose of α-pinene had a more significant impact on cognitive impairments than the 50 mg/kg dose. These findings are consistent with previous research, showing that intraperitoneal injection of 25 mg/kg of ketamine impairs memory and learning in rats (Li et al., 2020). Evidence suggests that α-pinene may be a valuable natural agent for treating or preventing amnesia and other neurodegenerative impairments related to learning, memory, and cognition (Lee et al., 2017). Research has also demonstrated that α-pinene treatment can ameliorate motor dysfunction and avoidance memory, accompanied by reduced brain MDA levels (Khoshnazar et al., 2020). Additionally, α-pinene has been shown to regulate the expression of proteins involved in acetylcholine synthesis and the antioxidant defense system, resulting in improved memory and learning (Wojtunik-kulesza et al., 2021, Weston-Green et al., 2021).

Impairment of mitochondrial function has been established as a cause of neurobehavioral abnormalities in animal models, leading to increased ROS production, DNA and protein oxidation, and lipid peroxidation (Ermakov et al., 2021) (kheradmand et al., 2018). Oxidative stress, defined as a shift in redox balance from physiological to pro-oxidant states, represents the failure of reparative molecular pathways to maintain cellular redox stability, ultimately resulting in oxidative damage (Perkins et al., 2020). The current study demonstrated that in the ketamine-induced mice model of SCZ, lipid peroxidation increased while TAC, GSH levels, and CAT and SOD activities decreased. However, α-pinene treatment reversed these changes in the hippocampal and cortical areas, enhancing GSH levels, antioxidant enzyme activities, and TAC while reducing MDA levels, a marker of lipid peroxidation. The 100 mg/kg dose of α-pinene increased CAT activity and GSH levels more effectively than the 50 mg/kg dose. Several studies have concluded that oxidative stress in ketamine-induced SCZ model of mice is associated with reduced SOD and CAT activities, a decrease in total antioxidant status, and an increase in lipid peroxidation products such as MDA (An et al., 2018, Ermakov et al., 2021). SCZ patients exhibit abnormal oxidative stress parameters with reduced total antioxidant levels. Oxidative stress is a crucial biomarker in SCZ's pathophysiology, clinical course, symptomatology, cognitive symptoms, and responses to antioxidant treatment (Lin and Lane, 2019). Reductions in TAC have been identified as a significant contributor to redox imbalances in SCZ (Ermakov et al., 2021).

The activation of the mammalian target of the rapamycin (mTOR) pathway by ketamine enhances the hippocampal translation of brain-derived neurotrophic factor (BDNF). Furthermore, ketamine rapidly releases glutamate by blocking NMDA receptors and activating AMPA receptors, inducing calcium influx through L-type voltage-gated calcium channels (VDCC) and releasing BDNF from synaptic vesicles (Réus et al., 2015).

Another study using a ketamine-induced mice model of SCZ reported decreased GSH levels, reduced CAT and SOD activities, and increased lipid peroxidation levels (Ben-Azu et al., 2018b, Sampaio et al., 2018). An investigation in an Alzheimer's disease model demonstrated that treatment with α-pinene increased GSH levels and decreased MDA levels in the brain (Khan-Mohammadi-Khorrami et al., 2022). In the investigation of the in vitro neuroprotective property of α-pinene in oxidative damage induced by H_2_O_2_ in PC12 (rat pheochromocytoma) cells, it was found that this monoterpene led to an increase in the protein level of redox-regulating enzymes such as CAT, SOD, glutathione reductase, and glutathione peroxidase. Generally, Porres-Martínez et al. supplied evidence for the valuable therapeutic effect of monoterpenes like α-pinene on the oxidant-antioxidant balance and demonstrated that this compound is a preventive agent for the treatment of neurodegenerative diseases caused by oxidative stress. (Porres-Martínez et al., 2016). The treatment of mice induced by acute toxoplasmosis with a-pinene for 14 consecutive days significantly reduced the serum level of MDA as a principal marker of lipid peroxidation and one of the oxidative stress consequences (Kharazmkia et al., 2022). Similarly, studies have shown that α-pinene, acting as an antioxidant, reduces lipid peroxidation and enhances CAT and SOD activities in the cortical and hippocampal regions in a rat model of focal ischemic stroke (Khoshnazar et al., 2019). In a study on isoproterenol-induced myocardial infarction in rats, α-pinene treatment significantly decreased lipid peroxidation and improved CAT and SOD activities and GSH levels (Zhang et al., 2020). Furthermore, another study revealed that α-pinene treatment safeguarded the nervous system from pathological damage by preventing oxidative stress, enhancing TAC, inhibiting lipid peroxidation, and increasing SOD and CAT activities. When administered orally, treatment with α-pinene at doses of 100 and 200 mg/kg for 14 days improved motor dysfunction and avoidance memory, accompanied by reduced MDA levels in the brain and blood (Khoshnazar et al., 2020).

Dopamine levels in the brains of ketamine-treated mice were elevated in both cortical and hippocampal areas. Treating α-pinene at a 100 mg/kg dose normalized dopamine levels in both regions, while α-pinene at 50 mg/kg only normalized cortical dopamine levels. Other studies have suggested that alterations in dopamine metabolism contribute to oxidative stress by generating radicals such as superoxide and hydrogen peroxide. Moreover, brain dopamine levels have been increased in mice models of ketamine-induced SCZ (Yadav et al., 2018a, Chatterjee et al., 2012). Based on previous research dysfunction in the striatal and prefrontal cortex (PFC) is implicated in the positive and negative symptoms of psychosis resulting from acute and chronic ketamine administration, likely driven by hyperdopaminergic and glutamatergic signaling (Vines et al., 2022). Moreover, previous evidence suggests that acute administration of ketamine in rodents is linked to a significant increase in dopamine levels in the cerebral cortex, striatum, and nucleus accumbens (Forti et al., 2023). As well, Lorrain et al. demonstrated that administering ketamine, a non-competitive NMDA receptor antagonist, increased glutamate and dopamine levels in dialysate obtained from the medial PFC of male rats (Lorrain et al., 2003). Metabolites of dopamine can also interact with Fe^3+^ and Cu^2+^ to generate ROS, further intensifying oxidative stress. NMDAR antagonists have been linked to increased dopamine and glutamate release, leading to a lack of cortical inhibition. As corroborated by recent research, dysfunctions in glutamate, dopamine, and GABA neurotransmissions are central to the pathogenesis of SCZ (Ermakov et al., 2021, Venkataramaiah et al., 2021). Research has shown that the accumulation of dopamine in the cytosol can lead to neurotoxicity and oxidative stress. ROS are produced naturally in dopamine degradation pathways. Cytosolic dopamine increase can exacerbate oxidative damage and have adverse effects on neuronal survival (Masoud et al., 2015, Kita et al., 2009, Chen et al., 2008). NMDA receptor antagonists, including ketamine, have been shown to impair GABAergic neuron function, resulting in increased firing of pyramidal cells and subsequent stimulation of dopaminergic neurons. Based on available evidence, NMDAR antagonists promote excessive glutamate release, leading to enhanced stimulation of dopaminergic neurons due to the disinhibition of glutamatergic neurons (Kokkinou et al., 2018). ROS disrupts neuron cell membranes, causing disruptions in dopamine neurotransmission, which plays a crucial role in the pathogenesis of SCZ. Increased dopamine synthesis has been associated with heightened prodromal symptoms, and hyperactivation of dopamine is evident during acute phases of SCZ and following psychological stress (Ermakov et al., 2021).

The findings from the current investigation indicate that oxidative damage, cognitive deficits, anxiety, depressive-like behaviors, and dopaminergic dysfunction are critical features of the ketamine-induced SCZ model. Therefore, α-pinene, a potent antioxidant, may offer therapeutic benefits in ameliorating the impairments associated with the ketamine-induced model of SCZ. The research provides compelling evidence that α-pinene possesses notable antioxidant properties and can improve anxiety- and depressive-like behaviors, cognitive deficits, and dopaminergic dysfunction in the ketamine-induced model of SCZ.

## 5 Conclusion

In conclusion, the current study, in conjunction with other research, provides conclusive evidence that α-pinene significantly exhibits neuroprotective effects in the hippocampal and cortical regions of ketamine-induced SCZ model of mice. Based on these investigations, α-pinene emerges as a potent antioxidant and a promising therapeutic option for ameliorating ketamine-induced impairments, including anxiety and depressive tendencies, cognitive deficiencies, and oxidative stress.

## Funding

This research did not receive any specific grant from funding agencies in the public, commercial, or not-for-profit sectors.

## CRediT authorship contribution statement

**Akbar Hajizadeh Moghaddam:** Study design, writing of manuscript. **Fatemeh Malekzadeh Estalkhi:** Project administration, Data collection, manuscript writing. **Tabarek Ahmed Hasan:** manuscript writing. **Sedigheh Khanjani Jelodar:** Data Analysis, methodology. **Mohammad Karimian:** methodology. **Soroush Farhadi-Pahnedari:** Project administration, Data collection.

## Declaration of Competing Interest

The authors declare that they have no known competing financial and personal relationships with other individuals or institutions that could affect their investigation reported in this paper.
